# Framing stigma as an avoidable social harm that widens inequality

**DOI:** 10.1177/00380261221150080

**Published:** 2023-03-20

**Authors:** Michelle Addison

**Affiliations:** Durham University, UK

**Keywords:** class, drugs, health inequality, social harm, stigma

## Abstract

This article discusses the social harms arising out of stigma experienced by people who use drugs (PWUD), and how stigmatisation compromises ‘human flourishing’ and constrains ‘life choices’. Drawing on Wellcome Trust qualitative research using in-depth, semi-structured interview data (*N* = 24) with people who use heroin, crack cocaine, spice and amphetamine, this article firstly provides insight into how stigma is operationalised relationally between people via a lens of class talk and drug use predicated on normative ideas of ‘valued personhood’. Secondly, it turns to how stigma is weaponised in social relations to keep people ‘down’, and thirdly, it shows how stigma is internalised as blame and shame and felt deeply ‘under the skin’ as ‘ugly feelings’. Findings from the study show that stigma harms mental health, inhibits access to services, increases feelings of isolation, and corrodes a person’s sense of self-worth as a valued human being. These relentless negotiations of stigma are painful, exhausting and damaging for PWUD, culminating in, as I argue, everyday acts of social harm that come to be normalised.

## Introduction

Stigma kills (NHS Addictions Alliance, 2021). The impact of stigma on the lives of the most vulnerable, underserved and underheard in society is gaining traction across sectors like health and social care, housing, criminal justice and education (Addison et al., 2022; Bambra, 2018; Black, 2020; Marmot, 2018; NHS Addictions Alliance, 2021). Stigma has interested academics because of its conceptual power to understand permutations of social and health inequality amid a diversity of experiences at both an individual and population level (Hatzenbuehler et al., 2013; Room, 2005; Scambler, 2018; Tyler, 2013b). This article adds to this rising tide of concern about inequality by drawing connections between stigma and avoidable social harms that are experienced relationally by vulnerable people – in this case, people who use drugs (PWUD). It takes as its focus and intellectual contribution, the intersections of embodied subjectivity through a lens of class and gender in synergy with the ‘deviant’ practice of drug use. Building on the pioneering work of Tyler (2020), Scambler (2018) and Hatzenbeuhler (2017), this article begins by providing insight into how stigma is operationalised relationally between people via a lens of class talk and drug use that is predicated on normative ideas of ‘valued personhood’ (Skeggs, 2011; Skeggs & Loveday, 2012). Secondly, it turns to how stigma is weaponised in social relations to keep people ‘down’ (Hatzenbuehler et al., 2013; Phelan et al., 2008; Scambler, 2018), and thirdly, it shows how stigma is internalised as blame and shame and felt deeply ‘under the skin’ (Kuhn, 1995), conceptualised here as ‘ugly feelings’ (Ngai, 2007). In doing so, I argue that class and gender, combined with drug use, form a toxic mechanism of stigma that is weaponised against marginalised and minoritised individuals, generating invisible social harms that are unacceptably normalised (Hatzenbuehler et al., 2013; Tyler, 2020). By adopting a social harm approach, it is possible to gain insight into how stigma practices compromise ‘human flourishing’ and constrain ‘life choices’ (Pemberton, 2016).

In the year ending 2020, it was reported by the Office for National Statistics drawing on the 2021 Census that 1 in 11 people aged 16-59 years old had taken a drug in the UK, with men nearly twice as likely as women to use drugs. Drug use increases to 1 in 5 people for those aged between 16 and 24 years old. Whilst the Census survey collects self-reported data on drug use, findings from the ONS suggest that people living in areas of high deprivation and in low-income households (total household income of less than £10,400) were more likely to have taken a drug in the last year, with cannabis being most prevalent. Approximately 1.1 million people (16-59 years) have taken a Class A drug (e.g. heroin, powder cocaine, ecstasy) in the last year, and 2.1% of adults (16-59 years) use drugs more than once a month and are categorised as frequent users (Office for National Statistics, 2020). It is important to bear in mind that these data refer to the period before the global coronavirus pandemic; global statistics show that drug use increased for frequent users during this time (Office for National Statistics, 2020).

In writing about the experiences of PWUD, it is important to recognise the heterogeneity of this group and that social harm is experienced unevenly; it is vulnerable PWUD that are more disposed to incur greater harms due to a multiplication effect from toxic, synergistic and compounding factors that are contextual, relational and intersectional in society (Addison et al., 2022; Bambra et al., 2021; Black, 2020). As Pemberton (2016, p. 3) writes, ‘[d]epending on the resources and social capital we are able to draw on, our ability to respond to specific social harms can differ significantly, which in turn means that harms can have contrasting impacts on a person’s life chances’. In the UK, there were 4561 deaths related to drug poisoning – half of these were related to heroin usage, which is more prevalent in socially deprived areas. The North East of England continues to have the highest rates of death related to drug misuse and it is has some of the most deprived districts in the UK (Ministry of Housing Communities and Local Government, 2019). These particular PWUD often have multiple complex needs, experience the relentless pressures of poverty, inhabit oppressed intersections of identity, and suffer widening health inequalities due to a lack of investment in improving the social determinants of health in their area (Bambra, 2018). As such, the focus on stigma as social harm amongst vulnerable PWUD in this article is particularly timely because it highlights not only a hardening of public attitudes but also a heightening of social and political governance of what is considered ‘acceptable citizenship’ during a period of extreme economic hardship and widening inequalities (Wacquant, 2008).

To understand stigma it is helpful to see it as a *verb*: that is, something that is done *between* people (Addison et al., 2022). As Scambler (2018, p. 767) argues, ‘attributes are neither creditable nor discreditable *in* themselves’ but are inscribed with stigma relationally. It is not possible to treat stigma as something which is material and objective, to say ‘*here is stigma*’, because stigma operates at a symbolic level – stigma has *meaning* and becomes apparent through its effect on people. Stigma functions as a symbolic system that is operationalised in social relations between people via mechanisms that attach value (or not) to intersecting identity attributes and (so-called deviant) practices (Scambler, 2018; Tyler, 2020).

Our social relations are structured by hegemonic norms, rules and conventions within a particular society and so are structuring of our social interactions with others (Bourdieu, 1990). Knowing how to *be* in the world, and what attributes and practices have value, amounts to knowledge capital that is accrued over time (Addison, 2016; Allen, 2007; Bourdieu, 1990). Bourdieu (1984/2010; 1990) writes how we are all ‘*born into the game*’ that is already taking place. A person’s position in the game and how they play it depends on their *habitus*, capital and the structures of the field; taken together this generates practices. Bourdieu describes this interrelationship as: [(*Habitus*)+(capital)] + field = practices (Bourdieu, 1986). As Maton notes, practice is contingent on a person’s *habitus* and the nature and amount of capital that they hold, as well as their position with the field (Maton, 2008). We learn how to act by acquiring knowledge of the ‘*rules of the game*’, and we use this knowledge to help navigate social relations (Addison, 2016). The way people interact depends on how a person is positioned in society (status) and their understanding of prevailing social relations. As such, we act differently around certain kinds of people, and in certain spaces and places as we attempt to navigate stigma (Addison, 2016). However, these social relations are profoundly unequal, shaped by structures of power that connote value, prestige and status (Addison, 2016; Scambler, 2018; Tyler, 2020), which I explore more in the remainder of this article.

## Research design

This study is qualitative in design to understand the everyday lived experiences of being in the world, and voices of ordinary, underheard and underserved, individuals (Allen, 2007; Back, 2007). This research utilised semi-structured in-depth interviews with 24 people (12 men, 11 women, 1 transgender; aged between 20 and 50 years old; majority white British sample) to understand how ‘a liveable life’ is made possible (Back, 2007) amongst PWUD. I acknowledge from the outset that the whiteness of the study sample necessarily represents only certain communities and is a limitation of the study. The population of the North East of England has the largest white British demographic (93.6%) in the UK (HM Government, 2022); purposive sampling aimed to achieve greater diversity and inclusion of minoritised voices in the study, however the majority white British sample achieved is a reflection of the population composition in the North East. As such, I am mindful that my study is not revealing of minoritised voices pertaining to intersections of race and ethnicity.

People living in already deprived circumstances are more affected by growing social inequalities than others, and as a consequence are more exposed to health-related problems (Bambra, 2016). As such, this research was located in the North East of England because it has the highest rate of drug-related deaths in the UK and a high density of deprived areas (Office for National Statistics, 2020). The research used a combination of purposive and snowballing sampling to try to include a diverse range of voices, adopting inclusion criteria that focused on people who used heroin, cocaine or crack cocaine, spice (novel psychoactive substance) or amphetamine as their primary drug of choice, although a range of frequency of drug use was acceptable. Participants had to be over 18 years old to participate and were recruited into the study via social media, leaflets, posters, and a range of established contacts within voluntary and third sector organisations who acted as gatekeepers and helped to establish contact with interested persons. Sound ethical practice (approved by university ethics board – submission ref: 17304) was adhered to: participation in the study was voluntary and all interested persons were given the opportunity to look at an information leaflet about the study and ask questions. All participants were provided with a £10 shopping voucher. Fieldwork occurred before and during the global Covid-19 pandemic (2020–2021) meaning that a combination of face to face (before Covid-19) and online/telephone (during Covid-19) interviews were conducted to mitigate the risk of contracting the virus.

## Main findings

### Class talk and drug use

Previous studies about class have highlighted just how difficult a subject it is to talk about (Addison, 2016; Finch, 2001; Reay, 2005; Sayer, 2005). There has been much research that has demonstrated that class is more than a materialist lens on the world (Reay, 2005; Sayer, 2002; Skeggs, 1997) and that socio-economic status (SES) does not adequately capture a person’s lived everyday experiences of class (Sayer, 2005; Skeggs, 1997). My discussions with PWUD give insight into a painful ‘psychic landscape of class’ (Allen, 2007; Reay, 2005). Class is loaded with moral overtures (Sayer, 2005), a means of judging people (Bourdieu, 1984/2010) based on dominant classificatory schemas (Addison, 2016; Bourdieu & Wacquant, 2013; Skeggs, 2011; Tyler, 2015), a lens in which to claim or inscribe a valued/valueless personhood (Skeggs, 2011), and mobilised as a mechanism of stigma (Tyler, 2020) that reaffirms power dynamics whilst also doing social harm (Pemberton, 2016; Pemberton et al., 2017). ‘Class talk’ is difficult precisely because it involves knowledge of the ‘game’ – that is, how to *be* in the world, and *how* to ‘play the game’ (Addison, 2016). Participants in this study variously did ‘class talk’ through identification, disidentification, negation and misrecognition of symbolic value systems. These participants anchored their working-class identification in where they were from, how they looked, status and employment:

‘Oh where are you from?’ ‘Redwood Park’, ‘Oh, right, what’s it like living over there?’ as though they were looking down on us. (Sarah, 39 years old)

I feel like, maybe someone in a lower class, living on a rough estate. (Alan, 20 years old)

. . . your status and that, and your job and that. (Tony, 26 years old)

. . . like people don’t want you about, you walk in a shop they automatically think you’re a shoplifter just because of the way you look or whatever. Yeah, there’s definitely a fine line between what’s acceptable in people’s eyes and what’s not. (Kev, 41 years old)

Turning to drug use, class and other intersections of identity like race and gender frequently led to complicated discussions – participants knew that being seen as working class, female and a PWUD combined mechanisms of stigma with toxic effects that were challenging to navigate. These intersections coupled with stigmatised ‘deviant’ practices were imbued with value distinctions (Bourdieu, 1984/2010). This is discussed by Theo and Tony in the following excerpts:

You’ve got politicians that are getting caught out for using drugs and you’ve got people at the bottom end of the scale that are using drugs [. . .] The only difference I would say would be the drug that you use – if you’ve got loads of money it’s going to be cocaine, it’s going to be the *fancy* drugs, but others at the bottom end of the scale – it’s heroin. (Theo, 39 years old)

I’ve always been common. Yeah, and when I’ve got on drugs and that, I feel worse now. Yeah, looked down on. (Tony, 26 years old)

Conversations with participants showed that drug type and available capital (social, economic, cultural) played a crucial role in determining how they were seen, and subsequently mitigated stigma harms. Many of the participants highlighted that stigma was not weaponised against middle class PWUD in the same way it was towards them. As Lawler and Payne write, those in positions of power and privilege are able to mobilise capital to advance their positioning further (Lawler & Payne, 2018); in this study this meant circumventing stigma as social harm. In contrast, others who were already oppressed and marginalised found mobilisation of valued capital to offset the harms of stigma extremely challenging:

. . . you’re just at the bottom of the barrel. (Kev, 41 years old)

I’m just back to being a druggy. Which isn’t a good thing is it? Like everybody just classes druggies as like lower than them. (Samantha, 36 years old)

. . . a lot of the time you judge someone as soon as you meet them and you can’t help doing that, can you? Everyone does that, don’t they? Not judging as in, like, ‘Oh, you’ll be this,’ or, but, like, you’ll probably think, ‘Oh, he’s scruffy,’ or he’s. . . and you might not be nasty about it, you might not say, ‘Oh, you’re a scruffy xxxx, I don’t like you, you look like you’ve just got out of bed,’ or anything, but you might just like, you can’t help judging. . . it’s just natural, isn’t it? (Tony, 26 years old)

What is revealing here is the sensitivity to being judged by others based on embodied subjectivity and ways of being in the world (Addison, 2017; Addison et al., 2022). There is an awareness that people can be valued differently *through* social relations between people, and that stigma mechanisms (Tyler, 2020) operate to make some better and ‘above’ those who are othered. Elsewhere, Allen (2007) has written about the pain and suffering experienced by PWUD; he shows how marginalised individuals can be framed negatively as ‘problematic’ through dominant power structures that serve to perpetuate inequalities. Allen argues that the identity of a ‘problematic drug user’ is constructed through awkward responses to the ‘body-subject’ of a person who uses drugs, and repeatedly through service responses (medical, justice) that individualise and pathologise the use of drugs, whilst simultaneously overlooking social context and an understanding of what *‘being in the world’* means to these people. This sense of being judged, and stigmatised, is further captured in my discussion with Hannah, who gives insight into the intersectional impact of gender – that is, being a woman and a mother, combined with class and drug use:

I just felt stigmatised that I was an addict and that I’d fallen pregnant do you know what I mean, I just feel like – there’s so much more on women and so much more. (Hannah, 35 years old)

In contrast, Alan talks about his current precarious class position (student-class) as temporary – he is a student who partakes in drug use (cocaine and MDMA) weekly to enhance his experience of dance culture, as well as improve his mental health and wellbeing at university (Addison et al., 2021).

I’d say my standards of living are obviously quite a bit lower, but not to the point where it would make me think I was a lower class. . . I think it’s just general. . . I know it doesn’t exist, but I’d say *student class*. (Alan, 20 years old)

Drug use is a financially costly activity, and he shares with me that he dislikes having no money and living in a dirty student flat; however, Alan is able to draw on his middle-class economic, social and cultural capitals (Bourdieu, 1990) to reassert his position, negate negative connotations of drug use, and make legitimate claims to a valued person-hood. Elsewhere, Jack describes feeling outside of a class regime. Indeed, what is revealing here is the sense that in order to be judged as belonging to a class, one has to be *seen*, and Jack powerfully describes the feeling of being overlooked because he is not considered to be a *human being* (Tyler, 2013b, 2020). Jack feels dehumanised by people in society:

People in society. People like. . . the classes. They don’t class me. Like someone with a lot of money – they don’t even look at me or class me as *a human being*. (Jack, 43 years old)

Whilst some more middle-class participants I spoke to were able to ‘play the game’ and mobilise various capital at their disposal to treat stigma as temporary or invalid, others were less fortunate, occupying marginalised positions in society, and thus encountered the full and harmful effects of stigma. I now discuss the weaponisation of stigma (Scambler, 2018) through social relations in the next section.

## Weaponising stigma in social relations to keep people ‘down’

The weaponisation of stigma is a means of keeping people ‘down’ (Hatzenbuehler et al., 2013; Phelan et al., 2008; Scambler, 2018). Phelan et al.’s research highlights how stigma can function to keep people ‘down’ (exploited), ‘in’ (conforming to norms) and ‘away’ (excluded from society) so that power structures remain unchallenged, and inequality persists to benefit dominant groups. Elsewhere, Hatzenbeuhler et al. (2013, p. 4) write, ‘Stigmatizing others enables people to achieve the ends they desire’. Scambler (2018) helpfully conceptualises the mobilisation of stigma in this way as *weaponisation* – a practice that inflicts social harm to retain power. I now turn to examples of this in my study.

### Everyday experiences of stigma

Participants talked about stigma that they had to deal with everyday just from taking up space or interacting with people. The pejorative word ‘smackhead’, a highly stigmatising label for a person who uses heroin (Wakeman, 2016), was discussed:

. . . you get them people walking down the street shouting ‘Smack head’, whatever else, you just put up with it don’t you, it’s one of them things. I mean you get. . . I’ve been stabbed in the neck, I’ve been jumped on by groups of lads just because I do what I do, not because I’ve done anything to them in particular, but they just feel they’re better than me. (Kev, 41 years old)

I was coming out, what do you call it, the food kitchen, food bank place, and it’s only daft young ones and that but some still look down on you [. . .] It’s all right if it’s just me and them there, but when they shout [smackhead] in front of people. . . (Sarah, 39 years old)

. . . people’s comments – sort of like *smack heads* and all. . . you hear all these comments. I don’t know, I just feel like these other classes look down on us [feels] just absolutely crap to be honest. (Jack, 43 years old)

These people described how this all-encompassing identity inscription took them by surprise and generated painful feelings of shame and humiliation. The normalisation of these everyday occurrences is troubling. This sense of being ‘put down’ again and again, and being kept down, as an everyday experience, is captured next by Jack, who highlights the relentless acts of stigmatisation that he endures, and we get a sense of how disorientating and unfair this experience can be:

What people get from being horrible to people and. . . I just do not get that mindset. I don’t. I’m sorry and that like, but I just do not get how people can enjoy seeing people suffering and causing people distress. I just don’t understand how –

M – Has that been a big part of your life, then?

It’s been a massive part of my life. It’s just like, “F**k”. They always put us down, put us down and put us down and put us down [. . .] They say it to put you down, so you then. . . it ruins your day, or you get to feel sh*t about yourself or you feel completely judged. (Jack, 43 years old) Being judged in this way is strongly linked to an affective landscape – of feeling disgusted (sh*t) and this impacting, indeed ruining, entire days. There is a wide literature base that attests to the damage prolonged periods of stress and isolation – in this case triggered through stigmatisation – can have on a person’s health (Bambra, 2016; Marmot, 2018; Shildrick et al., 2012). As scholars working in this area have noted, stigmatisers can utilise many intersecting mechanisms to be effective at achieving their aim (Hatzenbuehler, 2017; Link et al., 2017), and these stigma mechanisms can be constantly adapted. Stigmatisation, directed in this way, ensures that a distinction is drawn between a person of ‘value’ and someone regarded as ‘value*less*’: ‘*I always thought heroin users were the lowest of the low*’ (Chelsea, 43 years old). Haven discussed the temporary and fragile nature of being seen ‘legitimately’ as a person of value, and shared how they felt that their acceptance was contingent on maintaining and performing their recovery status:

. . . when I was in active addiction, it was a huge barrier, absolutely massive and I think the acceptance I get now is very conditional on me being in recovery and being in recovery the right way, you know? I need to be inspiring and have wise thoughts and if I’m even just a messy human being who happens not to do drugs anymore, that’s not good enough. So, it does feel like a very conditional acceptance where a lot of people will be like, ‘Oh my God, you’re so brave’, if you say you’re a recovering heroin addict but if I said, ‘Okay, I’m going to do some more heroin’, that would change very, very quickly. So, that can feel quite fragile at times as well. (Haven, 30 years old)

Having knowledge capital to embody and perform the ‘right’ kind of person in recovery, and to be able to ‘play the game’ to gain and sustain acceptance from others, was crucial (McGovern et al., 2021). This balancing act of performing recovery in the right way can be incredibly stressful and demonstrates an affective understanding that stigma mechanisms are still operating and require constant negotiation, resistance and refusal.

### Negotiations, resistance and refusals

Being able to navigate mechanisms of stigma was challenging for several participants. The ability and capacity to be able to do this work was contingent on: knowing how to ‘play the game’ and fit in; what resources and ‘capital’ participants had available to them; and an ability to mobilise these resources. Almost all participants expressed that they could sense when stigmatisation was happening in relations between themselves and others, but several described feeling very exposed and vulnerable, unable to challenge this value exchange. In my discussion with Tony it appears at first that he is impassive to the stigmatising judgements of others. However, as this talk unfolds, we see that ‘not caring’ is an expression of disengagement arising out of an imbalance of power to control for mechanisms of stigma (‘*there’s nothing I can do about it*’). This power imbalance between Tony and the stigmatiser is highlighted in his recognition of a forceful inscription of judgement (‘*they’ll say I’m doing it*’):

I don’t really give a fu*k anymore, to be honest. Everyone already knows what I do, so they’re going to think it anyway, they’re going to say it anyway. Even if I’m not doing it, they’ll say I’m doing it. It does get to me, don’t get us wrong, but there’s nothing I can do about it. (Tony, 26 years old)

In the below excerpt Jack also calls attention to the unfairness of mechanisms of stigma, which serve to erase any personhood and treat him like a ‘wasted human’ (Tyler, 2013a). Jack seeks to reframe the situation and *re*humanise PWUD, entreating wider society to link PWUD with embodied histories around trauma, abuse and loss. Jack frames PWUD as ‘*victims*’ – this is not an easy status for him to claim, given the normalisation of ‘ideal victimhood’ (Christie, 1986) in wider society as being generally white, female and ‘respectable’, and the complexity of PWUD occupying both victim/victimiser status in different moments. Jack plays on this and suggests that PWUD are victims of *society*, referring to multiple social problems relating to poverty, violence, abuse (aligned to social determinants of health), and the hardening of public opinion towards PWUD.

. . . people think, ‘Oh, heroin users, they’re just smack heads. They just go out robbing people.’ Heroin users. . . Heroin users are often people who’ve been abused, ex-alcoholics and people who’ve been through trauma who have lost everything: they’re victims of society. Drug users are *victims of society*. (Jack, 43 years old)

This is echoed in my discussions with Wendy, who also calls for the rehumanising of PWUD as whole persons who have had a complicated life history, tangled up with drug use. Stigma here is experienced through affect – instead of *contempt* for a PWUD, Wendy appeals for compassion and understanding, and to undo the social harm experienced through stigmatisation. She recognises that mechanisms of stigma enmeshed in social relations (judgements) can be so encompassing and reduce a person’s entire identity to having no value.

. . . nobody knows how to deal with it and you’re just stigmatised and labelled, so everybody thinks you’re just this one, collection of things, but you’re not. Everybody that’s a tortured soul of addiction, they all have something that’s triggered it off for them. I think more now I’m inside of the fence, it means I’m a lot more compassionate and a lot more slow to judge. . . (Wendy, 42 years old)

Similarly, Hannah discusses the retraumatisation she experiences in case review meetings with her social worker and wider social care team. She talks about feeling stigmatised and judged by professionals and being reduced to the case notes contained within her file and review documents.

. . .you just think, ‘Can’t. . . them people are going to read that without seeing the bigger picture or getting to know who I am or why I done these things’. And I think ‘Yeah, you *are* reading about child protection’. (Hannah, 35 years old)

Small acts of resistance, even to speak a thought out loud, emerged in our discussions. Jack recognises the judgements of others and how this is stigmatising; he powerfully disidentifies with this positioning of his self and refuses to be made abject by claiming his subjectivity (‘*I’m*’).

*I’m not* the person they f*cking see me as. (Jack, 43 years old)

Some participants, like Sarah, negotiated mechanisms of stigma by retaliating, expressing anger and confronting stigmatisers.

. . . but they don’t like it when they get the tables turned on them, when you say things back about them. (Sarah, 39 years old)

Whereas others in this study, as in the case of Haven below, felt able to resist the harmful effects of stigma (albeit knowing stigmatisation was occurring) by paying no heed to the value that was being ascribed to their personhood. Haven was however able to mobilise other economic capitals (Bourdieu, 1990) to mitigate the power imbalance created in social relations and assert a valued personhood.

I have experienced stigma, I have experienced judgement, but it’s never overly phased me, I’m not someone who at any stage of my life has been overly concerned with other people’s opinions [. . .] I wouldn’t care if all the doctors thought I was a liar and that I was full of diseases if they didn’t have the power to control my access to care. (Haven, 30 years old)

For Haven, stigma was a pragmatic problem that they encountered that generated physical and financial harms that they had to navigate: for instance, preventing access to essential resources (e.g. medical prescriptions, healthcare) (Chang et al., 2016). Some participants, particularly women, attempted to ‘play the game’ and tried to avoid being read through mechanisms of stigma based on class and drug use. The extant literature that highlights the complexity of gender performances, and the costs of getting it wrong, is compelling in this area (Delamont, 2001; Ettore, 2007). Carrie and Hannah both recognised that being stigmatised was costly to their status and devalued their personhood. They were acutely aware of ‘conductors of value’ (Tyler, 2015) associated with class and drug use and how they are perceived by others, such as trying to be discreet when sharing Universal Credit status to claim a prescription, trying to distance oneself from past drug practices, and dressing in a certain way.

I don’t know, it’s not being a snob, it’s just a bit of pride. When I go for my prescription, I don’t even like to say that I’m on Universal Credit. I’m not one to be. . . I know there’s no shame in that respect, but I don’t know. (Carrie, 43 years old)

. . . I think when you’re stigmatised most of your life and known as a heroin addict [. . .] I’ll say, ‘I pay this much for this’, and just. . . I think it maybe gives me a little bit more importance. [. . .] I do, I believe that that’s where. . . trying to live up to things. . . being an addict for all them years and not feeling part of society, where you know, if you’ve ‘got this or you’ve got that’ then you might feel part of it. I don’t feel I am. . . part of society to be honest, probably just a bit more important than I was, do you know what I mean? (Hannah, 35 years old)

To resist stigma, Hannah talks about making visible high-cost items of jewellery and clothing to assist her in claiming status and to strengthen a sense of belonging in society – although she laments that despite this work, she still does not feel she fits in.

## Ugly feelings: Shame and blame

Participants discussed how stigma made them feel and how this related to their past/present drug use. Having painful feelings, and being overwhelmed by them, was cited by the majority of people as being a key motivator to continue and/or increase their drug use (Addison et al., 2021). There was some discussion whereby participants wanted to experiment and ‘push the limits’ (edgework) of their lived experiences (Pennay & Measham, 2016) through drugs. For most in this study however, using drugs helped to change how they were feeling about themselves and their situation. Some participants expressed a need to block out unwanted feelings. Others wanted to experience more positive feelings, aside from feeling low, and used drugs to curate a confident and optimistic mentality, although recognising that this was somewhat ‘artificial’ and temporal.

. . . just hate feeling, it blocks everything out because. . . and I think because we’re that scared to start feeling stuff and like. . . Like I said this morning, emotions, what do you do with them and when you’ve been on drugs from such a young age, what do you do with all of these emotions that we’ve ran from for all them years. (Hannah, 35 years old)

When I was on the cocaine, right, I had to have it every morning to make me feel okay. (Nancy, 40 years old)

What was largely absent from most of these discussions was talk of the structural causes (Bambra, 2018; Marmot, 2010, 2018) linked to the way these people were feeling about being stigmatised. Instead, an individualising language of shame and blame permeated participants’ reflections – some expressed shame for *being* a person who had used drugs and knowing that they occupied a stigmatised position:

. . . it’s classed as something that’s degrading. So, like they call you [smackhead] to put you down. So, when the more people do that, the more it starts getting to you. And the more you start thinking right, well that’s *what I am*. (Samantha, 36 years old)

Shame is a huge driving force behind addiction. Guilt you can process, but shame is a lot deeper and it’s about what you think of your value, and I think when you live in a system where some people are seen as having more value than others. (Haven, 30 years old)

Ngai conceptualises blame and shame as ‘ugly feelings’ (Ngai, 2007). Ngai describes *ugly* feelings as experientially negative ‘in the sense that they evoke pain or displeasure’ (2007, p. 11). For Ngai, ugly feelings like ‘envy’ and ‘disgust’ are saturated with ‘socially stigmatizing meanings and values’ (2007, p. 11). They are different from other feelings like anger or joy, and can be difficult to even recognise in ourselves, let alone discuss with others. According to Ngai, these ‘ugly feelings’ are organised by ‘trajectories of repulsion rather than attraction’ and involve ‘processes of aversion, exclusion and negation’ (2007, pp. 11–12). Similarly, Sayer (2005) has also discussed the structural embeddedness and necessity of shame to ensure the reproduction of inequality. In the following excerpt, Carrie identifies stigma mechanisms in social relations (feeling judged) and how this gives rise to her feelings of shame. Knowing that she cannot change anything in her history, she discusses feeling stuck with guilt and this exacerbates the shame she feels. Carrie powerfully gives insight here to the corrosive effect shame can have on a person’s sense of worth and value – as something that literally ‘eats away’ at one’s sense of self.

I think they make me feel a bit ashamed. But that’s me, that’s because I’m sort of relating things to how I used to be. [. . .] I think pretty much everyone has a guilt about something. But to be ashamed of yourself is just like, it’s unhealthy, do you know what I mean? It’s not good for you because what’s happened has happened and you can’t change it. You need to not have that emotion *eating away* at you. (Carrie, 43 years old)

Similarly, Jack talks here about how he can sense being judged through how someone speaks to him and their body language:

I’ve self-medicated because of how f*cking low and destroyed I’ve been. . . I’ve felt about people. The body language and the way they act and the way they spoke to us. They really made us feel horrible and I don’t *deserve* this. (Jack, 43 years old)

Participants not only internalised shame for inhabiting a stigmatised identity, they also expressed shame for perceived harms they had done to others through their drug use.

I’ve done some really bad things to get the money for gear. I burgled my mum’s house, for f*ck’s sake! What kind of person does that? [. . .] I do feel that I’m the lowest of the low. My mum forgives me and she understands, she read up on heroin using. . . she disowned me for about a year, she didn’t want to, know me. . . (Chelsea, 43 years old)

. . . if you speak to the women who’s had their kids removed, or maybe put themselves in situations, there’s a lot of shame and guilt that maybe they just don’t want to ever face up to you know. (Hannah, 35 years old)

Many participants talked about feeling this ‘shame sanction’ arising out of stigmatisation which ‘subjects people to public humiliations’ (Tyler, 2020, p. 47). This symbolic act of stigmatisation signals, as Tyler puts it, a form of power that is literally inscribed onto the body and the whole identity of the person (2013b). It is intended to exclude and repel – generating a feeling of disgust (*abjection*). Tyler notes that disgust tends to invoke ‘consensus’, which is a powerful regulatory and stigmatising mechanism (2020). So, to position someone (or community) as disgusting is a form of ‘stigma-craft’ that helps to legitimate marginalisation and exclusion from mainstream society (Tyler, 2020).

### Blame: Self-stigmatisation

Participants adopted a frame of reference around responsibility and accountability that is often embedded in health behaviour interventions necessary to access healthcare and prescriptions, as well as language deployed in recovery and abstinence programmes. Whilst these programmes do much to provide immediate and sustained support to a PWUD, and reduce harm arising out of drug use, language adopted can be extremely individualising and can render structural inequalities invisible. Tyler draws on David Harvey’s body of work and describes this rhetoric of the ‘reflexive individual’ championed through neoliberalism as ‘an ideology which aims to restore and consolidate class power, under the veil of the rhetoric of individualism, choice, freedom, mobility and national security’ (2013a, p. 7). She goes on to state that ‘what characterises neoliberal states is the creation of “wasted humans”‘, which she argues is reproduced and perpetuated through social deprivation, labour precariousness and ‘heightened stigmatisation’ (2013b, p. 7). In my study, participants’ talk of agency and accountability frequently positioned individuals as ‘reflexive agents’; in the case of PWUD, this neoliberal responsibility rhetoric can translate to harmful negative talkback as participants became compelled to turn a harmful and stigmatising lens inwards and blame themselves, rather than look at structural and contextual factors. A number of participants discussed responsibility rhetoric, expressing shame and guilt for their drug use:

Well I’ve hardly any teeth left for starters and just basically screwed my life up because before my little sister and my older sister died I was speaking to them before I started the drugs and then once they found out I was on them they didn’t want nothing to do with me or anything [. . .] The only person to blame is myself, there’s nobody to blame for it but myself. (Alan, 20 years old)

[shame] is coming from a place of. . . that maybe we don’t deserve that. (M – Don’t deserve what?) – *Equality*, to be the same as everybody. (Hannah, 35 years old)

This practice of blaming and shaming oneself is particularly intensified in this discussion with Hannah as ‘responsibility’ becomes mapped onto ideals of motherhood.

If you’re lucky enough to still have something to fight for because you know, you’d probably put yourself right. If you don’t, you think I do deserve it, the kids have gone, this has gone so you’d keep on using with that guilt and shame. (Hannah, 35 years old)

### Mental health and coping mechanisms

Much research around the multiplicative effects of living in poverty show how stress can feel relentless and is damaging to health (Marmot, 2017; NHS Addictions Alliance, 2021; Pemberton et al., 2016; Public Health England, 2017; Wilkinson & Pickett, 2009). To cope with this everyday experience of extreme poverty some participants in this study described how they would use drugs to numb stress, stabilise their mental health momentarily, and to experience a positive feeling. This is captured in my discussion with Haven below:

Financial and security can cause so much stress and worry as well. It might not sound like a rational reaction that you can’t afford to keep a roof over your head so you spend what money you’ve got on drugs but it takes the edge off that anxiety, it’s an immediate fix for a long-term problem that you don’t have the solution to. [. . .] I think, it’s so much harder for people to get some kind of stability with mental health and recovery if they’re worried that they’re going to come home and find they’re being evicted from their home or they’re going to come home and the electricity has been cut off, or they’re going to come home and there’s no hot water. You’ve got that constant *fear* and constant *threat* to your safety and security. So how are you going to stabilise your mental health when you’re under constant threat? (Haven, 30 years old)

Being stigmatised had real ramifications for PWUD. It was linked to deteriorating mental health and negative self-talk, self-harm and suicidal ideation, as well as prolonged periods of isolation which have been related to poor health outcomes and health inequalities (Hatzenbuehler, 2017; Link et al., 2017). Jack shares how he withdrew from social interaction, reducing his social networks, which he compares to being imprisoned. This is also echoed by Ravi:

. . . people pass comment like as they do, and they don’t realise how damaging that is. That’s put me in. . . it’s kind of imprisoned me. It took my confidence away from us and I didn’t want. . . I don’t want anything to do with people anymore. (Jack, 43 years old)

I’ve just been a loner, just I felt isolated, on my own, unable to do anything about things, it’s been difficult. (Ravi, 42 years old)

Stigmatisation can create a feedback loop in which PWUD experience prolonged and intensified periods of low mood, which can then lead to (continued) self-medication using illicit drugs to change how they are feeling (Addison et al., 2021).

The more drugs you take, the worse you look, the thinner you look, the worse you look, the worse you look after yourselves, the worse people are going to *judge you*. It’s going to be worse. The more you do it, the worse it’s going to get. So, the drugs don’t really help. (Tony, 26 years old)

Although limited for space here, it is important to briefly highlight that many participants talked about mechanisms of stigma operating in interactions with service providers. This aligns to findings from NHS Addictions Alliance in which engagement with services and health outcomes of PWUD was linked to experiences of stigma in service interactions (NHS Addictions Alliance, 2021). Furthermore, Chang’s research in this area also shows how service users felt they had to mobilise ‘health capital’ in order to understand and navigate the healthcare system, and ‘perform’ the ideal and ‘deserving’ patient (Chang et al., 2016).

. . . for a lot of years I couldn’t get proper treatment for my legs because they just look at you, ‘You’re just a heroin user but that’s your own fault’, sort of. And you do get very negative things, I mean I got took into hospital because I’d OD’d and the nurses had stripped me off and thing. . . and as soon as, “It’s a heroin overdose” you just see their faces *change* and the way they sort of are working. . . (Jack, 43 years old)

I was a bit wary about going asking for help and that because I was like, ‘Oh, are they going to judge me?’ (Sarah, 39 years old)

Whilst I discuss the mobilisation of health capital elsewhere (Addison et al., forthcoming 2023), it is worth stating here that the social harm arising out of relational stigma can have physical health consequences: these participants were very sensitive to being judged and as a result, would miss appointments or avoid accessing the help that they needed altogether because stigmatisation was retraumatising.

## Reflections

This article presents findings that show how relentless negotiations of stigma are painful and damaging for PWUD, culminating in everyday acts of social harm that come to be normalised (Pemberton, 2007; Pemberton et al., 2016). Stigma is indeed felt ‘*under the skin*’ (Addison et al., 2022; Kuhn, 1995). Stigma unfairly corrodes a person’s sense of worth as a human being and impacts health, wellbeing and self-actualisation. Attributes and embodiments of class were marked out and stigmatised by the general public, health providers and between vulnerable groups in a myriad of ways that map onto subjectivity and practice, including: the way a person looked, where they lived, how they spoke, dressed, smelled, and what they *did* (e.g. use of particular kinds of drugs, in certain places and around certain people) were all given as examples of the way class ‘value’ became inscribed, recognised and mobilised as stigma. Class inscription was visceral when it came to these particular people who used drugs and was made even more complex at the intersection of gender – with working-class women experiencing forms of stigmatisation mapped on to expectations of ‘good’ mothering and respectable ways to do femininity (see also Skeggs, 1997). The intersection of class, gender and drug use, grounded in ways of *being* in the world, was utilised as a powerful and intricate mechanism of stigma, as described by Tyler (2020), and weaponised (see Scambler, 2018), to inflict harm perceived as normal in society.

The intersection of stigmatised classed and gendered subjectivities (although I remind the reader this is representative of a majority white sample) taken in synergy with stigmatised deviant practices (drug use) served to dehumanise people, generating a feeling of ‘*abjection*’. This experience of being made ‘abject’ – that is, to be made unhuman or valueless – highlights the invisible social harms that arise out of stigma. In this study, PWUD’s experience of being made ‘abject’ had deleterious impacts on mental health, how they accessed services, and added to a sense of isolation and exclusion from wider society. Therefore, it is important to recognise that stigma as social harm is unevenly experienced, impacts the most vulnerable, and exacerbates the widening of social and health inequalities.

Throughout this discussion, I have been interested in the question ‘*In whose interests does stigma serve?*’ What I hope has become clear is how mechanisms of stigma can be mobilised within social relations to structure interactions, maintain power structures and reproduce stubborn inequalities between and across sub-populations of vulnerable groups (Hatzenbuehler, 2017; Scambler, 2018; Tyler, 2020). Whilst these mechanisms of stigma can change over time, adapting and combining intersections of identity in synergy with various deviant practices and wider structural harms – just like an amoeba, the act of *weaponising* stigma remains constant (Scambler, 2018). As Tyler (2020) argues, weaponising stigma is a *craft* that can be operationalised at a structural level through state violence, and as I have tried to show here – in everyday interactions between people, as a means to preserve privilege and maintain inequalities. Phelan et al. (2008) describe this struggle to retain power through mechanisms of stigma as a way for dominant factions to keep marginalised subgroups ‘down’ (exploited), ‘in’ (conforming to norms) and ‘away’ (excluded). It is important that we recognise stigma is a form of power that is all too often normalised and accepted, amounting to an ‘indifference to human suffering’ towards marginalised and minoritised people (Pemberton, 2004). We must see stigma as social harm if we are to challenge and respond to it as social problem. Mechanisms of stigma are deeply problematic and compromise ‘human flourishing’ and choice (Pemberton et al., 2016), and are only starting to be recognised as a cause of concern by policymakers and practitioners (NHS Addictions Alliance, 2021).

This article is a call to action: mechanisms of stigma, by their very design and the way society is organised, unfairly and unjustly impact the most vulnerable and marginalised people by reproducing power structures, serving the interests of the privileged, and thus adding to the widening of social and health inequalities. It is vital that we recognise that stigma is a social harm, that it widens inequalities, and it is *avoidable* and *preventable*. Therefore, more needs to be done to recognise and respond to the social harms arising out of stigma experienced by marginalised and minoritised people.
